# Profiling of LINE-1-Related Genes in Hepatocellular Carcinoma

**DOI:** 10.3390/ijms20030645

**Published:** 2019-02-02

**Authors:** Tomoyuki Honda, Md. Arifur Rahman

**Affiliations:** 1 Division of Virology, Department of Microbiology and Immunology, Osaka University Graduate School of Medicine, Osaka 565-0871, Japan; arifur@nstu.edu.bd; 2 Department of Microbiology, Noakhali Science and Technology University, Noakhali 3814, Bangladesh

**Keywords:** hepatocellular carcinoma, hepatitis B virus, tumorigenesis, LINE-1, DNA damage, retrotransposition

## Abstract

Hepatocellular carcinoma (HCC) is a prime public health concern that accounts for most of the primary liver malignancies in humans. The most common etiological factor of HCC is hepatitis B virus (HBV). Despite recent advances in treatment strategies, there has been little success in improving the survival of HCC patients. To develop a novel therapeutic approach, evaluation of a working hypothesis based on different viewpoints might be important. Long interspersed element 1 (L1) retrotransposons have been suggested to play a role in HCC. However, the molecular machineries that can modulate L1 biology in HBV-related HCC have not been well-evaluated. Here, we summarize the profiles of expression and/or activation status of L1-related genes in HBV-related HCC, and HBV- and HCC-related genes that may impact L1-mediated tumorigenesis. L1 restriction factors appear to be suppressed by HBV infection. Since some of the L1 restriction factors also limit HBV, these factors may be exhausted in HBV-infected cells, which causes de-suppression of L1. Several HBV- and HCC-related genes that interact with L1 can affect oncogenic processes. Thus, L1 may be a novel prime therapeutic target for HBV-related HCC. Studies in this area will provide insights into HCC and other types of cancers.

## 1. Introduction

Hepatocellular carcinoma (HCC) is a prime public health concern that causes almost 90% of the primary liver malignancies in humans. HCC is the sixth most common cancer and is the fifth leading cancer in males and ninth most common cancer in females [1,2]. The mean 5-year survival rate of HCC patients was found to be between 25% to 60% [3]. Despite recent advances in treatment strategies, there has been little success in improving the survival of HCC patients.

The most common etiological factor of HCC is hepatitis B virus (HBV) infection [4]. Worldwide, over 50% of HBV patients with chronic HBV infections progress to liver cirrhosis (LC) and 70% to 90% of them eventually develop HCC [5,6]. At present, there are approximately 257 million HBV carriers, and 887,000 deaths were reported due to the HBV-related complications including LC and HCC in 2015 [7]. During HBV infection, the HBV partially double-stranded DNA genome (relaxed circular DNA, rcDNA) is repaired and converted into covalently closed circular DNA (cccDNA) that can act as a template for the synthesis of viral transcripts including pre-genomic RNA (pgRNA) [8,9,10,11]. pgRNA is reverse-transcribed to generate rcDNA for viral replication [10]. The HBV genome encodes at least four genes, *pre-core*/*HBc*, *Pol*, *HBs* and *HBx* [10,12]. Hepatitis B e antigen (HBeAg) and hepatitis B surface antigen (HBsAg) are HBV-specific antigens derived from pre-core/HBc and HBs, respectively.

The incidence of HCC or HBV persistent infections may vary with geography, race, age, and sex. Co-infection with hepatitis C virus (HCV), a family history of HCC, alcohol intake, HBV genotype C, and core promoter mutations are considered to be risk factors for HCC [13,14,15,16,17,18,19]. For example, there is an increased risk of developing HCC in adult males and chronic hepatitis B patients with cirrhosis who contracted HBV in early childhood [3]. Patients who are both HBsAg- and HBeAg-positive have a 6-fold risk of developing HCC than those who are only HBsAg-positive [20]. However, the molecular mechanisms of how HBV contributes to HCC tumorigenesis are not fully understood.

Long interspersed element 1 (LINE-1 or L1) is a non-long terminal repeat (LTR) retrotransposon that comprises ~17% of the human genome [21]. L1 can retrotranspose to new genomic loci in a “copy-and-paste” manner [22,23]. Most L1s are truncated and therefore defective for retrotransposition activity, whereas ~100 copies remain competent [22,23]. Therefore, active retrotransposition of L1 can be a major source of endogenous mutagenesis in humans, which may contribute to genomic instability and tumorigenesis [24,25]. Consistently, L1 upregulation in cancer has been frequently reported [26,27,28,29]. In addition, L1 de novo insertions can alter gene expression [30,31], which also potentially contributes to cancer development [32,33,34]. Among cancers, HCC is considered to be the one in which L1 might be involved for the following reasons [31,32,34]. Firstly, the majority of L1 de novo insertions have been detected in cancers [35]. Secondly, HCC is an extraordinarily heterogenous cancer, apparently because of genomic instability [36,37]. Thirdly, endogenous L1 retrotransposition has been demonstrated to activate oncogenic pathways in HCC [31]. Fourthly, several L1 chimeric transcripts with host or viral genes are found in hepatitis virus-related HCC [38]. Finally, it has been demonstrated that L1 retorotransposition is a common feature of HCC caused by various mechanisms [34]. Based on these, we have speculated that HBV may modify L1 biology and thereby potentiate HBV-infected hepatocytes to develop HCC [32,33].

In this regard, we discuss the potential molecular linkages between HCC, especially HBV-related HCC, and L1. Starting with a brief introduction of the biology of L1 retrotransposon, we review the expression profile of L1-related genes in HCC and/or their roles in HBV-related HCC. Then, we illustrate the possible interactions between HBV- and HCC-related genes and L1. An understanding of the possible molecular links between HCC and L1 might open up avenues for the development of novel therapeutic approaches for this disease.

## 2. Long Interspersed Element 1 (L1)

Approximately half of the human genome consists of retrotransposons with or without LTRs. Among these, L1 is a unique non-LTR retrotransposon, because some of them are still capable of mobilization in the human genome [22,23]. L1s contain a 5′ untranslated region (UTR), two open reading frames (ORFs) that encode two proteins, ORF1p and ORF2p, and a 3′ UTR with a polyadenylation signal. ORF1p is an RNA-binding protein with nucleic acid chaperone activity, which is required for L1 retrotransposition [39]. ORF2p is responsible for endonuclease and reverse transcriptase activity [22,23]. L1 reverse-transcribes and integrates into new genomic loci by target-primed reverse transcription (TPRT) [40]. During TPRT, L1 creates a nicked DNA strand, which serves as a primer for reverse transcription, using the endonuclease activity of ORF2p. Environmental factors, such as chemicals, oxidative stress and infection, are capable of affecting L1 retrotransposition [32,41,42,43]. For example, human immunodeficiency virus type 1 (HIV-1) infection enhances L1 retrotransposition and increases the amount of L1 DNA [44]. HIV-1 Vpr and Vif proteins play a role in activation of L1 retrotransposition [44,45]. Therefore, it is reasonable to speculate that HBV may activate L1 retrotransposition.

Active L1 retrotransposition can potentiate oncogenic processes in various ways. As mentioned above, since L1 causes insertional mutations, any potential disruption of tumor suppressor genes by L1 retrotransposition could contribute to the development of tumors. L1 de novo insertions can affect the expression of nearby genes and the genes into which they have inserted [30,31]. If an L1 insertion occurs close to an oncogene or a tumor suppressor gene, the inserted L1 may increase oncogene expression or decrease the expression of tumor suppressor genes, thereby supporting tumor development. L1 provides preferential sites for genomic rearrangements [46], which may contribute to genomic instability that causes tumorigenesis. DNA strand-breaks produced by ORF2p during TPRT can also cause genomic instability. Occasionally, L1 retrotransposition creates new chimeric transcripts, which might also enhance tumor development [38].

## 3. L1-Related Genes in Hepatocellular Carcinoma (HCC)

**Figure 1:**
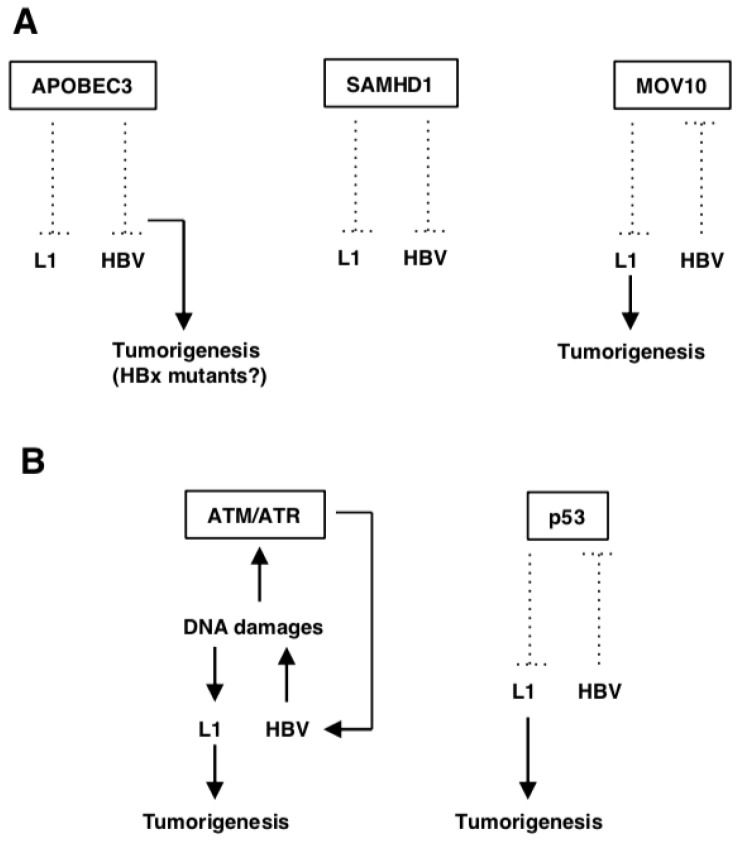
L1-related genes in HBV-related HCC. (**A**) Host defense genes against L1. APOBEC3s suppress L1 retrotransposition and HBV replication. Some APOBEC3s generate HBx mutants that cause gain of function, enhancing its oncogenic properties. SAMHD1 also inhibits both L1 retrotransposition and HBV replication. MOV10 is downregulated by HBV infection, which may upregulate L1 retrotransposition and accelerate tumorigenesis. (**B**) L1-related DDR genes. HBV appears to induce DNA damages, which can activate the ATM/ATR pathway, required for efficient HBV replication. On the other hand, HBV-induced DNA damages can potentiate L1 retrotransposition and cause genomic instability. HBV inactivates p53, which can activate L1 retrotransposition.

Many host genes are involved in L1 biology. Among them, we focus on two categories of genes, i.e., genes related to host defense and DNA damage responses (DDRs), which may potentially affect the oncogenic processes of HCC (Figure 1). In addition, we summarize L1 de novo insertions that may involve HCC development.

### 3.1. Host Defense Genes Against L1

Apolipoprotein B mRNA editing enzyme catalytic polypeptide 3 (*APOBEC3*), sterile alpha motif domain and HD domain-containing protein 1 (*SAMHD1*), and Moloney leukemia virus 10 homolog (*MOV10*) are three well-known genes that have been identified as host defense factors against HIV-1 and L1 [47,48,49,50,51,52,53]. Because HBV genome replications involve a reverse transcription step, similar to L1 or HIV-1, these factors also restrict HBV and might affect HBV-mediated tumorigenesis.

APOBEC3, a cytidine deaminase, whose family members inhibit L1 retrotransposition [51] reportedly hyper-edits the HBV DNA as well as inhibits HBV replication in vitro and in vivo [54,55,56,57]. APOBEC3s are incorporated into nascent HBV capsids, where they convert cytidine bases to uracil in newly synthesized DNA. This modification causes degradation of the modified HBV DNA or disruption of coding sequences by incorporating numerous G-to-A nucleotide mutations into the positive-strand of the viral DNA [54]. Thus, HBV could enhance L1 retrotransposition by competing with A3G restriction (Figure 1A). The APOBEC3B expression was up-regulated in a variety of cancers including HCC [58]. Furthermore, APOBEC3s play a role in the development of HCC during chronic HBV infection [54]. For example, some APOBEC3s generate HBx mutants that (especially the C-terminally truncated mutants) cause a gain of function, enhancing the colony forming ability and proliferative capacity of HBV-infected cells. As a result, the cells obtain a selective clonal growth advantage (Figure 1A) [59].

SAMHD1 restricts efficient viral cDNA synthesis by reducing the pool of dNTPs [60,61]. It can restrict DNA viruses and retroviruses including HIV-1 [62,63,64,65,66]. The depletion of cellular dNTP pools has been regarded as a key anti-viral mechanism of SAMHD1 [67]. Additionally, it also exhibits RNase activity that directly targets retroviral genomic RNA, blocking productive infection in a dNTPase-independent manner [68]. SAMHD1 also inhibits L1 retrotransposition by sequestrating the L1 ribonucleoprotein complex within stress granules [51] or suppressing L1 reverse-transcription (Figure 1A) [69]. In the HBV life cycle, SAMHD1 has no effect on covalently closed circular DNA (cccDNA) production or HBV gene expression, while it specifically inhibits the reverse-transcription step through the depletion of cellular dNTPs (Figure 1A) [70]. The full-length SAMHD1 acts as an anti-tumor factor by increasing the cell sensitivity to chemotherapy drugs [61]. Incorporation of exon-4 of SAMHD1 has been linked to a higher prevalence of HBV- and HCV-related HCC, which leads to an abnormal *SAMHD1* translation termination that weakens the anti-tumor activity of SAMHD1 [61,71]. Although exon-4 incorporation might be an indicator of hepatocarcinogenesis, the precise mechanism behind the occurrence of this insertion still needs to be studied.

MOV10, an interferon (IFN)-inducible RNA helicase, has very broad and potent anti-retroviral activity [52,72,73], which also suppresses L1 retrotransposition (Figure 1A) [53]. The overexpression of exogenous MOV10 resulted in an increase of HBsAg, HBeAg and HBV mRNA levels at a low dose, and a decrease at a high dose, while HBV DNA was unaffected. By contrast, knockdown of MOV10 could suppress levels of HBsAg, HBeAg and HBV mRNA, while it had no effect on HBV DNA [74]. These results suggest that an appropriate level of exogenous MOV10 supported HBV replication [74]. Patients with chronic hepatitis B produced lower levels of *MOV10* mRNA compared with healthy individuals [75]. Taken together, HBV may suppress the MOV10 expression, thereby enhancing L1 retrotransposition in infected hepatocytes (Figure 1A).

### 3.2. L1-Related DDR Genes

Ataxia telangiectasia mutated (ATM) and ATM-Rad3-related (ATR) are kinases activated by various types of DNA damages [76,77]. Activated ATM and ATR subsequently phosphorylate downstream substrates, Chk2 and Chk1, respectively, and p53. These effectors induce cell cycle arrest, DNA repair and/or cell apoptosis [76,77]. L1 retrotransposition is increased in ATM-deficient cells [78]. HBx activates the ATM-Chk2 pathway by inducing DNA damages [79]. Additionally, HBV infection triggers ATR-dependent DDRs and increases ATR and Chk1 phosphorylation levels [80]. Although the precise role of ATM and ATR in HBV replication is unclear, ATM-ATR kinase inhibitors suppressed HBV infection and replication (Figure 1B) [80]. Since L1 can retrotranspose into DNA damage sites in its endonuclease-independent manner [81], L1 retrotransposition may be enhanced by HBV-induced DNA damages (Figure 1B).

p53 is known to be a tumor suppressor protein encoded by the *TP53* gene, which is closely associated with HCC through regulation of cell differentiation, cell cycle and cell apoptosis [82,83]. p53 activation is crucial for DDRs, effective chemosensitivity and improvement of the HCC prognosis [84]. p53 has been demonstrated to limit L1 retrotransposition, through which p53 might restrict oncogenesis, at least in part (Figure 1B) [85]. *TP53* is mutated in more than 45% of HBV-related HCC and in 13% of HCV-related HCC [86]. Preferential mutation sites are located within the DNA-binding domain of p53, which reduces its binding affinity to responsive elements and therefore decreases expression of p53 target genes [87]. Although the molecular pathogenesis of HCC can involve the inactivation of the *TP53* gene [88,89], the absence of a *TP53* somatic mutation in the majority of HCC cases [90] suggests that the inactivation can be achieved by other mechanism(s), such as p14^ARF^ inactivation [91] or the amplification/overexpression of its specific inhibitors, MDM2 and MDM4 [92]. In the HBV infection context, HBx binds to p53, inactivating p53 transactivation, which may contribute to hepatocarcinogenesis (Figure 1B) [93,94,95].

### 3.3. L1 de novo Insertions

As described in Section 2, L1 de novo insertions can trigger oncogenic processes. L1 de novo insertions into or nearby tumor suppressor genes or oncogenes may affect gene expression, thereby promoting tumorigenesis. L1 de novo insertions are categorized into two types, i.e., germline and somatic insertions. Germline L1 insertions are generated by retrotransposition events in germline cells, which will contribute to all tissues of the individual. An example of germline L1 insertions contributing to tumorigenesis is those into the mutated in colorectal cancer (*MCC*) gene that are associated with downregulation of the *MCC* gene [31]. *MCC* is a gene that suppresses the oncogenic Wnt/β-catenin signaling pathway, which is frequently activated in HCC [96], suggesting that downregulation of MCC caused by L1 insertions can lead to oncogenesis.

L1 retrotransposition is usually suppressed in somatic cells. However, if somatic L1 retrotransposition happens, the insertion can fuel tumorigenesis. For example, a tumor-specific L1 somatic insertion is found at the transcriptional repressor suppression of tumorigenicity 18 (*ST18*) gene, a candidate oncogene in the liver, and the insertion activates *ST18* expression [31]. Because the expression of *ST18* is upregulated in several liver cancer cells and in tumors in a mouse-model for inflammation-driven HCC, and L1 insertion upregulates the expression of *ST18* [31], L1 can enhance tumorigenesis through the upregulation of *ST18* by an L1 de novo insertion to the *ST18* locus.

## 4. HBV- and HCC-Related Genes in L1 Biology

**Figure 2:**
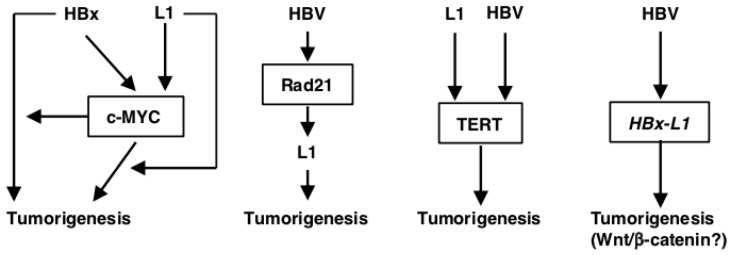
HBV- and HCC-related genes in L1 biology. HBx activates c-MYC, and HBx and c-MYC synergistically promote tumorigenesis. L1 de novo insertions were preferentially localized near the *c-MYC* gene, which may upregulate gene expression. L1 plays a role in genomic rearrangement in MYC-induced oncogenesis. Rad21 is upregulated in HBV-related HCC, which drives L1 expression. Upregulation of L1 may enhance L1 retrotransposition and thereby cancer development. HBV and L1 sequences are reportedly inserted into the *TERT* gene locus. The insertions upregulate the gene expression, which can affect tumorigenesis. The *HBx* sequence is inserted into L1 loci, which generates *HBx-L1* chimeric transcripts. *HBx-L1* functions as a non-coding RNA that activates the oncogenic Wnt/β-catenin pathway.

Many studies have reported hypomethylation of L1 loci in HCC and HBV infections [97,98,99,100]. L1 hypomethylation has also been linked to poor outcomes of HCC [97,98]. Recently, L1 activation was shown to be a common feature of hepatocarcinogenesis [34]. In this section, we discuss the links between particular HBV- and HCC-related genes and L1, HBV insertions and L1, and the roles of an HBV-L1 chimeric transcript (Figure 2).

### 4.1. Myc

A comprehensive review of all articles related to “HBV and HCC” published between 1973 and March 2018 has revealed that over 1300 host genes interact with at least one of the HBV proteins, the most frequent of which is HBx [101]. Of these, *GPT, AFP, ALB, IFNA1, TP53* and *MYC* have been discussed in at least 50 different articles regarding HBV-related HCC [101]. Among these 6 genes, *TP53* and *MYC* are oncogenes and L1 may play roles in *TP53*- and *MYC*-related oncogenesis. *TP53* is frequently mutated in HBV-related HCC, whose mutation/inactivation has been associated with a poor outcome of HCC, as described above (Figure 1B) [86,88,89]. *c-MYC* is a critical target gene that is often activated by HBx, which in turn accelerates the oncogenic properties of HBx [102,103]. In a transgenic mouse model, HBx alone has no direct pathological effects on developing HCC. The c-MYC/HBx-expressing transgenic mice rapidly produce tumors compared with c-MYC-expressing transgenic mice, illustrating that the synergistic effect of HBx and c-MYC accelerates the development of liver cancer. Moreover, the interaction between HBx and c-MYC stabilizes c-MYC by inhibiting c-MYC ubiquitination, which ultimately contributes to viral oncogenesis (Figure 2) [104]. Because c-MYC regulates a number of cellular genes that are involved in HBV-related HCC, *c-MYC* is not only an oncogene but also modulates the oncogenic activity in HBV-mediated HCC [105]. L1 reportedly participates in genomic rearrangement in MYC-induced lymphoma, supporting the idea that L1 also contributes to MYC-mediated oncogenesis (Figure 2) [106]. Furthermore, L1 de novo insertions were preferentially localized near the *c-MYC* gene [107], which may upregulate gene expression and contribute to oncogenesis (Figure 2).

### 4.2. CBX1, Rad21 and CENPA

Several gene expression profiling studies of HCC are reported previously. We reviewed them and found only four articles that provided full lists of genes that were differentially expressed in HCC [108,109,110,111]. Huang et al. applied RNA-seq technology to identify genes dysregulated in HBV-related HCC patients [108]. In the study, 1378 differentially expressed genes were reported, among which 808 was upregulated and 570 was downregulated [108]. Boyault et al. analyzed the gene expression profile of HBV-related HCC patients by genome-wide transcriptome microarray and identified 471 upregulated and 167 downregulated genes [109]. Gopal et al. carried out integrative transcriptome analysis of HCC patients, where 459 and 332 genes were shown to be upregulated and downregulated, respectively [110]. Okabe et al. analyzed genome-wide gene expression by microarray, and found that 165 were upregulated while 170 genes were downregulated [111]. We found 28 upregulated and 11 downregulated genes common in three of four studies. Among them, *CBX1*, *Rad21* and *CENPA* are supposedly involved in L1 biology.

The Chromobox 1 (*CBX1)* gene encodes a Chromobox protein homolog 1 protein, also known as HP1, which recognizes and binds histone H3 tails methylated at Lys-9, altering the chromatin structure and usually leading to epigenetic repression [112,113]. Additionally, *CBX1* can function as an oncogene [114]. The expression of CBX1 noticeably increased in HCC tissues compared with the non-tumorous ones [114]. High CBX1 expression was significantly associated with larger tumor size, poor tumor differentiation and tumor vascular invasion [114]. CBX1 overexpression promoted cell proliferation and migration, while the CBX1 knockdown showed the opposite phenotypes. CBX1 was proposed to be preferentially recruited to LINE sequences to form heterochromatin [115]. Thus, CBX1 appears to be a negative regulator of L1, whose contribution to HBV-related HCC is unclear.

Rad21 is a subunit of the cohesion complex [116]. Dysregulated expression of Rad21 is common in epithelial cancers [117,118] and its upregulation is associated with a poor prognosis [119]. Rad21 is reported to be enriched in the L1 promoter region and to drive L1 expression in human colorectal cancer [120]. Similarly, HBV may upregulate Rad21, which drives L1 expression and promotes L1 retrotransposition, resulting in the development of HCC (Figure 2) [120].

Centromere Protein A (CENPA) is a critical centromere-specific histone H3 variant that defines the neocentromeric chromatins [121]. Neocentromeres are ectopic centromeres that are able to assemble a functional kinetochore [122]. *LINE-1* RNA is proposed to serve as an epigenetic determinant in neocentromere formation [123]. Because neocentromeres were detected in at least two types of human cancer and aberrant hypomethylation, which causes L1 upregulation, contributes to hepatocarcinogenesis, upregulation of CENPA and LINE-1 synergistically triggers neocentromere formation, which may support chromosome segregation during the oncogenic proliferation of HBV-infected hepatocytes.

### 4.3. HBV Insertions

A key event in chronic HBV infection is the integration of the HBV sequences into the host genome and a total of 5331 integration events have been reported [101]. There is a direct relationship between the HBV DNA integration and HCC progression [101]. The most frequently integrated viral genes are *HBc* and *HBx*, while the most commonly reported integrated sites for HBV are the loci of the *TERT*, *MLL4*, *FN1*, *CCNE1* and *CCNA2* genes [101]. Except *FN1*, these integrations lead to the overexpression of the genes that have been implicated in HCC tumorigenesis [101].

The telomerase reverse transcriptase (*TERT*) gene is one of the most common genes associated with L1 de novo insertion [38,124]. In more than 90% of human malignancies including HCC, the *TERT* gene is reported to be activated by *TERT* amplification or *TERT* promoter mutations [125,126,127], leading to infinite proliferation of the cells [125]. *TERT* promoter mutations are thought to be a new biomarker to predict HCC as they are frequently found in premalignant lesions [128] and at an early stage of tumorigenesis, such as stage I HCCs [129]. Since aberrant expression of TERT is associated with tumor development, HBV and/or L1 sequence insertions in the proximity of the *TERT* locus may have a role in carcinogenesis by affecting the TERT expression (Figure 2) [125]. Consistently, the highest frequency of HBV integration is detected in the *TERT* gene, causing expression or reactivation of the *TERT* gene [130,131,132,133].

### 4.4. The HBx-L1 Chimeric Transcript

HBV integration into the intergenic region is also very common. Among the 9249 articles reviewed by Lee et al., 2789 were found to be integrated in intergenic regions [101]. Of these, 92 mapped to repeat sequences, of which 36 were on LINEs and 28 were in the L1 sub-family [101]. *HBx-L1*, a chimeric transcript of the *HBx* and *L1* sequences found in HBV-related HCC, was reportedly detected in more than 20% of HBV-related HCC and correlates with a poor outcome of HCC (Figure 2) [38]. *HBx-L1* knockdown reduces the migratory and invasive properties of HBV-positive HCC cells. *HBx-L1* overexpression confers growth advantage and promotes cell migration and invasion. The chimeric protein-coding potential of *HBx-L1* is not required for these effects, suggesting that *HBx-L1* may function as a long non-coding RNA that promotes HCC phenotypes. The expression of the *HBx–L1* chimeric transcript reduces the level of microRNA-122 (*miR-122*), increasing the activity of the Wnt/β-catenin signaling and inducing colony formation and cell cycle progression (Figure 2) [38,132,134,135].

## 5. Conclusions

**Figure 3:**
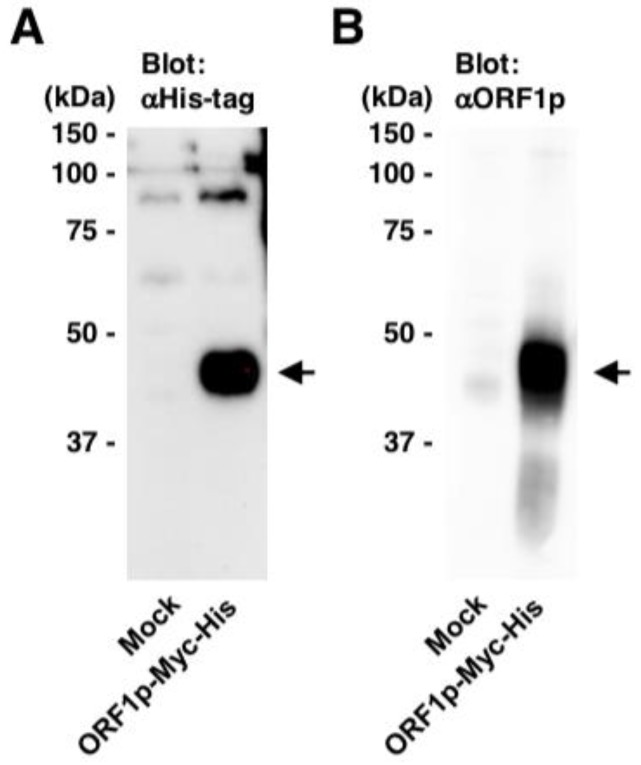
Generation of an antibody against L1 ORF1p. A rabbit antibody against L1 ORF1p (#18469) was generated using a synthetic ORF1p peptide [27–45 aa]. The homogenate of the cells transfected with pEF-Myc-His (Invitrogen) or pEF-ORF1p-Myc-His was subjected to western blotting using anti-His-tag (**A**) and anti-ORF1p (#18469) antibodies (**B**). Arrows, bands of ORF1p.

In HBV-related HCC, the expression and/or activation status of L1-related genes is altered, which may contribute to L1 activation and HCC tumorigenesis. To investigate this hypothesis, it is important to evaluate the L1 activation status in each cancer cell and surrounding non-cancer cells because HCC is highly heterogenous and L1 activity in each cell might be different [35,36,37]. Several antibodies against L1 (e.g., JH73 and AH40.1) have been established previously [136,137,138]. In addition, we have successfully generated a novel antibody against ORF1p (#18469) (Figure 3). These L1-specific antibodies might be a helpful tool for immunohistological analysis of HCC samples. Recently, ORF1p was shown to enhance the transcription factor activity of pregnenolone X receptor and to be involved in sorafenib-resistance in HCC cells [139]. The involvement of ORF1p in drug resistance in HCC further emphasizes the importance of evaluation of the ORF1p expression.

Although L1 is likely to be involved in the oncogenic processes of HBV-related HCC, it has not yet been demonstrated whether HBV indeed modulates L1 retrotransposition. HBV genes often contribute to the development of HCC. Among them, HBx is the best-studied viral protein in HBV-related HCC. HBx associates with various host factors in multiple cancer-related biological pathways. Therefore, HBx may be a candidate that modulates L1 expression and/or retrotransposition, by which oncogenic processes are potentiated. At present, we cannot exclude the possibility that other HBV proteins contribute to the regulation of L1 activity. Further investigation will be required for clarifying this point. Intriguingly, we have demonstrated that another oncogenic virus, Kaposi’s sarcoma-associated herpesvirus, can enhance L1 retrotransposition [140], which may highlight the importance of L1 in HBV-mediated oncogenesis.

Recently, we have also reported that capsaicin, a compound with anti-tumor activity, can suppress L1 retrotransposition [141]. This result suggests the possibility that some anti-tumor agents might exert their anti-tumor effect through the inhibition of L1 retrotransposition. Given that L1 plays important roles in HBV-related HCC tumorigenesis, L1 may be a novel prime therapeutic target for HBV-related HCC. Research in this regard will provide insights into HCC and other types of tumors.
